# Renal Lipotoxicity-Associated Inflammation and Insulin Resistance Affects Actin Cytoskeleton Organization in Podocytes

**DOI:** 10.1371/journal.pone.0142291

**Published:** 2015-11-06

**Authors:** Cristina Martínez-García, Adriana Izquierdo-Lahuerta, Yurena Vivas, Ismael Velasco, Tet-Kin Yeo, Sheldon Chen, Gema Medina-Gomez

**Affiliations:** 1 Departamento de Ciencias Básicas de la Salud, Área de Bioquímica y Genética Molecular. Universidad Rey Juan Carlos, Avda. de Atenas s/n, Alcorcón, Madrid, Spain; 2 Division of Nephrology/Hypertension, Northwestern University, Chicago, Illinois, United States of America; University of Valencia, SPAIN

## Abstract

In the last few decades a change in lifestyle has led to an alarming increase in the prevalence of obesity and obesity-associated complications. Obese patients are at increased risk of developing hypertension, heart disease, insulin resistance (IR), dyslipidemia, type 2 diabetes and renal disease. The excess calories are stored as triglycerides in adipose tissue, but also may accumulate ectopically in other organs, including the kidney, which contributes to the damage through a toxic process named lipotoxicity. Recently, the evidence suggests that renal lipid accumulation leads to glomerular damage and, more specifically, produces dysfunction in podocytes, key cells that compose and maintain the glomerular filtration barrier. Our aim was to analyze the early mechanisms underlying the development of renal disease associated with the process of lipotoxicity in podocytes. Our results show that treatment of podocytes with palmitic acid produced intracellular accumulation of lipid droplets and abnormal glucose and lipid metabolism. This was accompanied by the development of inflammation, oxidative stress and endoplasmic reticulum stress and insulin resistance. We found specific rearrangements of the actin cytoskeleton and slit diaphragm proteins (Nephrin, P-Cadherin, Vimentin) associated with this insulin resistance in palmitic-treated podocytes. We conclude that lipotoxicity accelerates glomerular disease through lipid accumulation and inflammation. Moreover, saturated fatty acids specifically promote insulin resistance by disturbing the cytoarchitecture of podocytes. These data suggest that renal lipid metabolism and cytoskeleton rearrangements may serve as a target for specific therapies aimed at slowing the progression of podocyte failure during metabolic syndrome.

## Introduction

Changes in lifestyle and dietary habits have raised the overall incidence of obesity [1]. These patients often suffer other comorbidities such as hypertension, heart disease, dyslipidemia [2] and renal disease. In recent years, several authors have shown that an excess of lipids and lipoproteins promotes renal disease progression. The lipids increase glomerular injury and tubulointerstitial fibrosis and accelerate the progression of renal disease in diabetic patients [3,4].

Hyperlipidemia associated with obesity also promotes glomerulosclerosis via mechanisms involving low-density lipoprotein (LDL) receptors in mesangial cells, renal oxidative damage, and increased macrophage chemotaxis by fibrogenic cytokines [5]. In this sense, high levels of triglycerides (TGs) and lipoproteins in plasma induce damage at the cellular level [6]. Published data report that HDL-bound cholesterol levels are positively associated with estimated glomerular filtration rate (eGFR) in healthy individuals of European and Asian ethnicity [7]. This assertion was supported by in vitro studies in cultured mesangial and tubular cells [8,9].

High levels of lipids are involved in the typical kidney lesions of obesity and induce direct podocyte damage [10]. Podocytes are dynamic cells mainly involved in the kidney filtration process and the maintenance of the glomerular filtration barrier (GFB), but they also participate in signal transduction mechanisms. Over the last decade, podocytes have become critically important due to the discovery of specific mutations in key physiological genes that lead to proteinuria [11–13]. Podocytes consist of a cell body, major processes, and foot processes. The foot processes with their actin cytoskeletons are attached to the glomerular basement membrane by adhesion proteins. Of note, foot processes from neighboring podocytes interdigitate with each other and form intercellular junctions called slit diaphragms (SD) [14]. The SD consists of several proteins such as nephrin, podocin, cluster of differentiation associated protein 2 (CD2AP) and zonula occludens-1 (ZO-1) which are closely linked to the actin cytoskeleton. This interaction influences cell motility and signaling pathways in the podocyte [15,16]. The SD is also required to control actin dynamics, response to injury, endocytosis, and cell viability. An altered expression or a physical disruption of these proteins plays a key role in foot process effacement, an unequivocal morphological finding in the development of proteinuria [17–19]. In addition, podocyte injury affecting the cytoskeleton or genetic alterations in the expression of certain cytoskeletal modulating proteins are observed in different forms of focal segmental glomerulosclerosis, in association with proteinuria and foot process effacement [20–23]. All these observations support the idea that regulation of the podocyte cytoskeleton is critical for sustained glomerular filter function [24,25]. In addition, the podocytes are unique among the glomerular cells in that they express all elements of the insulin signaling cascade, which also influences podocyte structure, function and survival [26].

Our previous studies performed in 4-week POKO mouse kidneys showed a faster renal disease progression than in the *ob/ob* mouse. POKO mice are hypertriglyceridemic, hyperglycemic and insulin resistant at an early age [27]. We observed changes in glomerular ultrastructure such as glomerular basement membrane thickening, extensive loss of podocyte foot process structure and a decrease in the density of inter-podocyte slit-diaphragm pores along the glomerular basement membrane. Furthermore, proteinuria was higher in POKO mice *vs*. WT mice. The podocyte structural organization was also altered, and nephrin and podocin gene expressions were decreased. These alterations may be caused in part by lipid accumulation at the glomerular level and the increased levels of ceramides and diacylglycerides.

In this study we investigated mechanisms and processes involved in lipotoxicity using cultured podocytes. The podocytes were treated with different doses of palmitic acid (PA) and/or cytochalasin D (CD), which prevents the polymerization of actin in the cells. We evaluated the role of the cytoskeleton in this type of injury. We conclude that palmitic acid accumulates in intracellular lipid droplets and promotes insulin resistance associated with inflammation, oxidative stress and endoplasmic reticulum stress in podocytes. We also found cytoskeleton rearrangements that were directly related to the previous processes driving podocytes to a final stage of apoptosis.

## Materials and Methods

### Cell culture and treatments

A conditionally immortalized mouse podocyte cell line was kindly donated by Dr. Peter Mundel (Harvard University, Cambridge, Massachusetts, USA), isolated from a transgenic mouse that has a thermosensitive variant of the SV40 large T antigen (H-2Kb-tsA58) inserted in its genome [28], the podocyte line proliferates at 33°C in the presence of mouse-γ-interferon (10 U/ml; Sigma-Aldrich) but becomes quiescent and differentiates when thermoshifted to 37°C in the absence of γ-interferon [15]. To induce differentiation, podocytes were maintained in RPMI 1640 supplemented with 10% FBS on Type I collagen (Sigma) pre-coated wells at 37°C without interferon-γ to suppress the T antigen. Differentiated podocytes were maintained for 14 days and a podocyte differentiation marker, synaptopodin, was identified by immunocytochemistry. Podocytes were grown to near confluence and serum-deprived before experiments. All experiments were performed after 12 h of serum starvation, and afterwards cells were incubated with control or treatment medium for 24 h unless otherwise specified. Passage numbers 19 to 27 were used for all experiments.

Palmitate treatment was performed as described earlier [29,30]. Briefly, 20% fatty acid-free BSA solution was heated to 37°C before the addition of a 100 mM palmitate (PA) (J.T.Baker: S874-05) stock solution dissolved in ethanol. The solution was heated to 37°C until clear and diluted with RPMI 1640 to give a final concentration of 5% BSA, 1% ethanol and 100, 500 or 750 μM palmitate [29]). The solutions were filter-sterilized (0.2 μm pore size) before being added onto the cells. The control for palmitate was 5% BSA and 1% ethanol, called vehicle (Veh). To perform the insulin stimulation assay, differentiated podocytes were maintained in serum-free medium for 18 h. Then the cells were incubated without or with PA (100, 500, 750 μM) for 24 h. Subsequently, cells were washed and insulin (Ins) was added for 5–10 minutes to a final concentration of 100 nM. To study the role of the cytoskeleton, we used cytochalasin D (Sigma-Aldrich®). Podocytes were pre-treated or untreated with 5 μM cytochalasin D for 2 h [23,31], before being incubated with 500 μM palmitate for 24 h.

### RNA preparation and qRT-PCR

RNA extraction and quantitative (q) RT-PCR were performed as previously reported [27,32]. The input value of the gene of interest was standardized to a housekeeping gene calculated using GeNorm reference gene selection kit (PrimerDesign Ltd, Southampton, UK). (N = 3 experiments). The specific sequences of primers used in this study are included in S1 Table.

### Protein extraction and Western blotting

The cells were washed twice with ice-cold PBS and scraped into RIPA buffer plus protein inhibitors. The protein concentration was determined as previously reported by Bradford MM et al., 1976 [33]. Proteins were separated on 10 or 12% SDS-PAGE and transferred to polyvinylidene difluoride filters. Membranes were blocked and probed with the following antibodies: anti-phospho-AKT (Thr308) (Cell Signaling), anti-total AKT (Santa Cruz Biotechnology, INC), anti-Tubulin (Sigma), anti-phospho-SAPK/JNK (Thr183/Tyr185), anti-total SAPK/JNK (56G8) (Cell Signaling), anti-phospho-p44/42 MAPK (Erk1/2) (Thr202/Tyr204) (E10), anti-p44/42 MAPK (Erk1/2) (137F5) (Cell Signaling), anti phospho-Ser307-IRS1, anti-total IRS1 (Millipore), anti-p65-NF-κB (C-20) (Sigma-Aldrich®), anti-Lamin B Receptor (abcam®) and anti-sXBP1 (R&D biosystems).

The protein band density was measured using the ImageJ 1.45 software (National Institutes of Health, Bethesda, MD). The amount of protein under control conditions was assigned a relative value of 100%.

### Oil Red O staining

The Oil Red O lipid staining was performed in podocytes as previously reported by Kinkel A. et al., 2004. First, cells were treated with PA and then fixed in 10% formalin. The staining time with Oil Red O reagent was 10 minutes. Oil Red O elution was performed by shaking with isopropanol (100%). The quantitative measurement was made by reading the absorbance at 500 nm of eluted dye with the spectrophotometer SPECTRA-Fluor Plus (TECAN, Austria).

### Scratch assay

Cell motility was assessed using a scratch assay modified from Lee et al. [34]. Prior to differentiation, cells were allowed to proliferate to achieve a confluence greater than usual (>70%). After serum starvation, culture media was removed and cells were subjected to two washes. A sterile 10 μl pipette tip was used to scratch the cell monolayer in 3 straight lines. Cells were washed twice in media to remove debris and 200× baseline images were taken with inverted microscopy (Nikon Epiphot). 24 h later, vehicle or PA (100, 500, 750 μM) treatment was applied. After that, cells were fixed in 10% formalin and stained with Crystal Violet dye. Images were taken by inverted microscopy. The number of cells that migrated into the scratch was counted in experimental *vs*. control conditions by Image J (v1.46; National Institutes of Health, USA). Viability of cells was quantified by a Crystal Violet method [35] in SPECTRA-Fluor Plus (TECAN, Austria). The results were expressed in arbitrary units normalized to the Crystal Violet viability data (N = 2 experiments, 8 fields/treatment).

### Measurement of superoxide production

The effect of PA on superoxide production in podocytes was determined by a fluorometric assay using dihydroethidium (DHE; Sigma, St. Louis, Mo) modified from a method described by Jiménez-Altayó et al. [36]. Dihydroethidium (DHE) is a fluorescent superoxide-anion probe (Beyotime, China). Following uptake by living cells, intracellular superoxide anions act on DHE to dehydrogenate it to ethidium that combines with DNA or RNA to generate red fluorescence DHE. DHE fluorescence occurs at excitation wavelength of 488 nm and an emission wavelength of 535 nm. Podocytes were seeded on coverslips in 24-well plates and treated for 24 h with vehicle or PA. Afterward, cells were exposed to 10 μM DHE dissolved in RPMI 1640 for 30 minutes at 37°C. To analyze if the fluorescence was increased by PA treatment, we assigned 1 point to vehicle fluorescence emission. Fluorescence was measured using a 20× objective of a fluorescence microscope (Axiophot Zeiss). Then we quantified the red fluorescence of all nuclei found per image (3 coverslips per treatment/3 fields/3 photographs of each) using image software AxioVision Software 4.6, Carl Zeiss (N = 3 experiments).

### Immunofluorescence

Podocytes on cover slips were fixed with 4% paraformaldehyde. After blocking, cells were incubated with anti-Paxillin (Calbiochem), phalloidin toxin-Rhodamine (Life Technologies™ R415), anti-p65-NF-κB (C-20) (Sigma-Aldrich®), anti-CHOP (Santa Cruz Biotechnology, Inc.) or anti-GLUT4 (Millipore). Secondary antibodies were FITC-conjugated. Some samples were incubated without the primary antibody to serve as negative controls. The nuclei were visualized using DAPI dye. Photographs were taken using an inverted fluorescence microscope (ECLIPSE 90i, Nikon Instruments Europe B.V.) or confocal microscope LSM710 (Zeiss, Germany). GLUT4 images of podocytes were scored by two independent blinded observers who scored at least 100 cells per condition for cytoplasmic or peripheral localization.

### Enzyme-Linked ImmunoSorbent Assay

IL-6 and MCP-1 concentrations were measured in media supernatant following the instructions of the commercial kits for Mouse IL-6 ELISA Sandwich (Fisher Scientific) and Mouse JE/MCP-1 ELISA Quantikine (R&D Systems). Absorbance was read in SPECTRA-Fluor Plus (TECAN, Austria) at the wavelengths as indicated by the kits. The results were expressed in arbitrary units normalized to the total amount of protein per well. (N = 4 wells/treatment; N = 2 experiments).

### Detection of Apoptotic Cells

To detect DNA breaks of apoptotic cells in situ, we used a Terminal deoxynucleotidyl transferase (TdT)-mediated Dig-labeled nucleotide Nick-End Labeling (TUNEL) method using ApopTag® Peroxidase in situ Apoptosis Detection Kit (Millipore). Cells were grown on a twenty four-chamber slide; fixed in 4% neutral buffered formaldehyde solution and rinsed with PBS. After treatment with 3% H_2_O_2_ at room temperature for 10 min, the cells were incubated with TdT enzyme for 60 min at 37°C. The Dig-labeled nucleotides incorporated into DNA breaks were detected by applying anti-Dig-POD and DAB. Finally, the nuclei were counterstained with methyl green.

### Statistical analysis

Results are expressed as mean ± SEM (standard error of the mean). Statistical differences and interactions were evaluated through a one-way or two-way factorial analysis of variance (ANOVA) using the Kruskal-Wallis test. When statistically significant differences resulted at the interaction level, Student’s t-test or Mann-Whitney test was carried out to compare the experimental data two by two. Differences were considered statistically significant at P<0.05. GraphPad InStat (GraphPad Software, Inc) was used.

## Results

### Intracellular lipid accumulation and lipid metabolism in podocytes treated with different doses of palmitic acid

**Fig 1:**
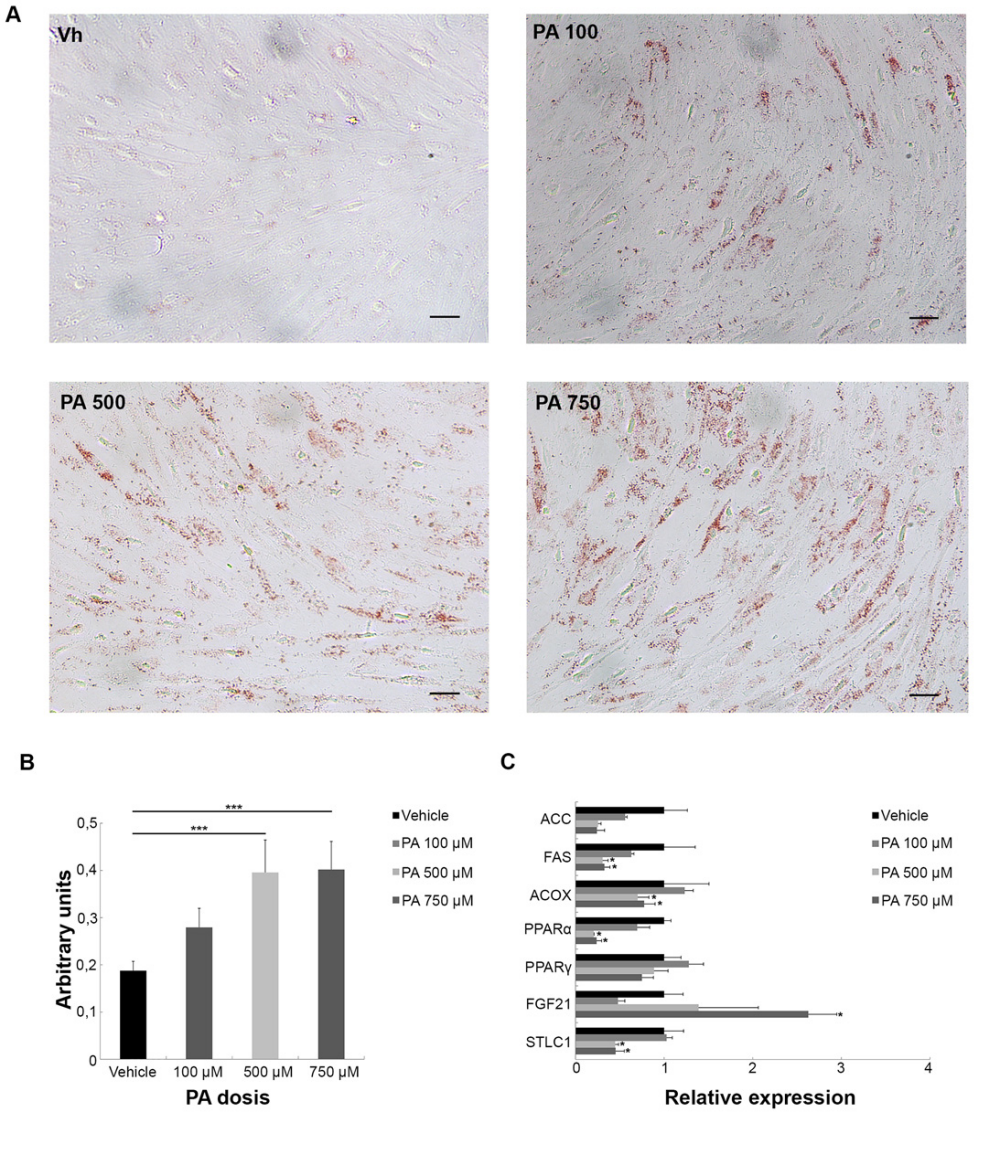
Intracellular accumulation of lipids and changes in lipid metabolism in PA-treated podocytes. (A) Representative Oil Red O staining in podocytes treated for 24 h with vehicle, 100, 500 or 750 μM of PA. (B) Colour quantification after dye elution (n = 3 experiments). Original magnification: 200x. (C) mRNA levels of genes related to lipid metabolism: *Fibroblast growth factor-21* (FGF21), *Serine palmitoyltransferase-1* (SPTLC1), *Acetyl Coenzyme A Carboxylase* (ACC), *Fatty Acid Synthase* (FAS), *Acetyl-CoA-Oxidase* (ACO), *peroxisome proliferator activated receptor alpha* (PPAR), and *peroxisome proliferator activated receptor gamma 1* (PPARγ1) in podocytes treated 24 h with vehicle, 100, 500 or 750 μM of PA. Data is expressed as mean ± SEM and normalized with GeNorm.*** p<0.001 PA *vs*. Veh.; ** p<0.01 PA *vs*. Veh.; * p<0.05 PA *vs*. Veh.

To assess whether podocyte treatment with PA causes intracellular lipid accumulation, we visualized total neutral lipid content by the Oil Red O technique (Fig 1). Fig 1A and 1B shows a significant accumulation of lipids in podocytes treated with 500 or 750 μM of PA compared with vehicle control.

Gene expression of enzymes involved in fatty acid synthesis such ACC and FAS decreased at 500 and 750 μM of PA compared to vehicle-treated podocytes (Fig 1C). In addition, mRNA levels of *Acetyl-coenzyme-A oxidase (Acox)* in podocytes treated with 500 or 750 μM of PA also significantly decreased, indicating a decrease in β-oxidation of fatty acids with high doses of PA. Similarly, *Pparα* mRNA expression significantly decreased in PA-treated podocytes at the same doses. Although *Pparγ2* levels were undetectable (data not shown), mRNA levels of *Pparγ1* showed the same tendency to be decreased in podocytes treated with high doses of PA. On the contrary, mRNA expression of Fibroblast growth factor 21 (Fgf21), a growth factor related to tissue lipid accumulation, was significantly increased in podocytes treated with a PA dose of 750 μM compared to vehicle. Finally, the expression of *serine palmitoyl transferase-1 (Sptlc1)*, involved in the initial step of ceramide synthesis, was significantly decreased in 500 and 750 μM of PA-treated podocytes.

### Palmitic acid treatment produced inflammation and insulin resistance in podocytes

**Fig 2:**
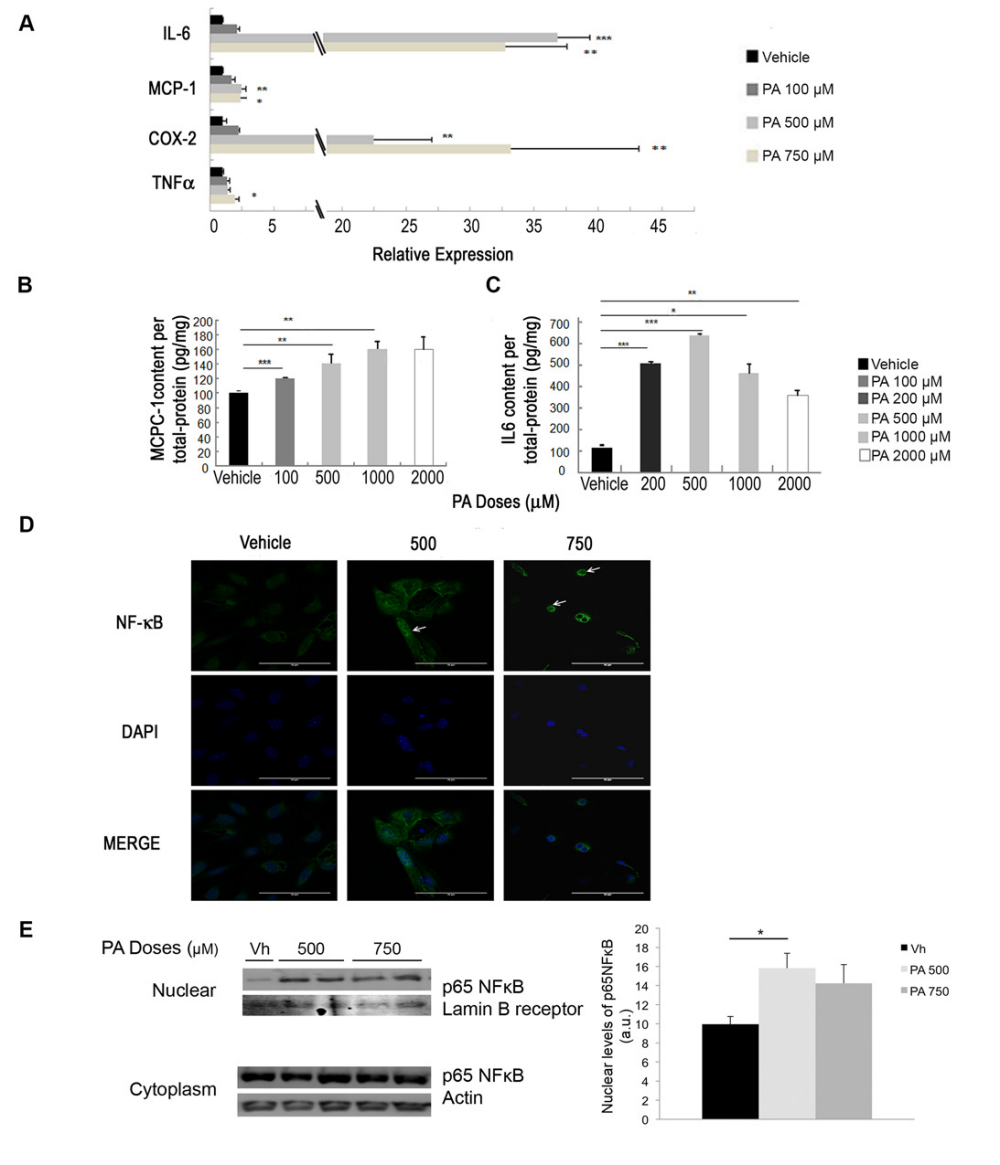
PA produces inflammatory effects in podocytes. (A) mRNA levels of inflammatory related genes: Interleukin-6 (IL-6), Monocyte chemoattractant protein 1 (MCP-1), Cyclooxygenase 2 (COX-2), Tumor necrosis factor (TNF-α) in podocytes treated 24 h with vehicle, 100, 500 or 750 μM of PA. Data is expressed as mean ± SEM and normalized with GeNorm; (n = 3 experiments). ELISA quantification in secreted media of podocytes treated 24 h with vehicle, 100, 200, 500, 1000 or 2000 μM of PA (n = 3 experiments) for: (B) Monocyte chemoattractant protein 1 (MCP-1) or (C) Interleukin 6 (IL-6). (D) Representative micrographs of the immunostaining for p65 NF-κB in podocytes treated 24 h with vehicle, 100, 500 or 750 μM of PA (white arrows at stained nuclei). Original magnification: 630×.(E) Representative immunoblot and quantification showing nuclear and cytoplasmic levels of p65 NF-κB from different doses of PA-treated podocytes. Nuclear levels were normalized to Lamin B Receptor and Cytoplasmic levels to Actin. *** p<0.001 PA *vs*. Veh.; ** p<0.01 PA *vs*. Veh.; * p<0.05 PA *vs*. Veh.

Analysis of the pro-inflammatory cytokine *IL-6*, a mediator of inflammatory response MCP-1 and the inducible *Cox-2* mRNA expression levels showed a significant increase in podocytes treated with 500 or 750 μM of PA compared to vehicle. *Tnf-α* mRNA levels in podocytes only increased significantly with 750 μM of PA treatment compared to vehicle (Fig 2A).

Following up on the gene expression results above, changes at the protein level were analyzed using ELISA. MCP-1 concentrations increased significantly in podocytes treated with PA concentrations from 100 to 1000 μM compared to podocytes treated with vehicle (Fig 2B). IL-6 protein levels significantly increased in podocytes at every dose of PA compared to vehicle (Fig 2C), showing the highest protein levels at 500 μM of PA.

NF-κB is one of the main transcription factors that control the transcription of inflammatory related proteins. Thus, we analyzed the influence of PA on p65 NF-κB translocation to the nucleus in podocytes. Fig 2D shows the increase of nuclear fluorescence (white arrows) in podocytes treated with 500 or 750 μM of PA compared to podocytes treated with vehicle. This was also confirmed by western blotting (Fig 2E), which shows an increased p65 NF-κB in the nuclear fraction upon treatment with PA.

**Fig 3:**
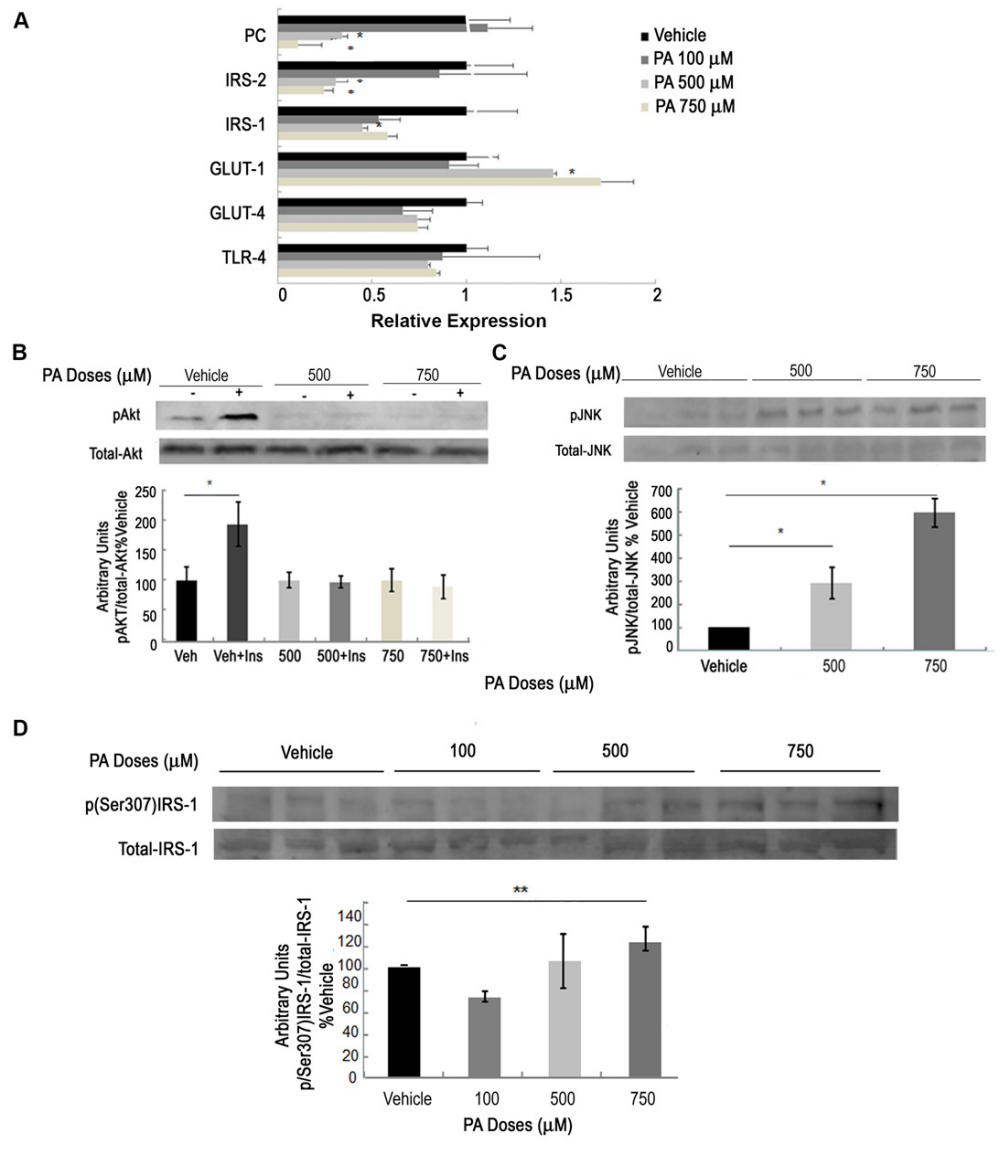
PA produces changes in glucose metabolism and insulin resistance in podocytes. (A) mRNA levels of genes related to glucose metabolism such as: *pyruvate carboxylase (PC)*, *insulin receptor substrate-2 (Irs-2)*, *insulin receptor substrate-1 (Irs-1)*, *glucose transporter-1 (Glut-1)* and *-4 (Glut-4)*, *and Toll-like receptor-4 (Tlr4)* in podocytes treated 24 h with vehicle, 100, 500 or 750 μM of PA (n = 3 experiments); (B) Representative immunoblot and quantification of pAkt(Ser473) protein extracts from different doses of PA-treated podocytes plus 100 nM insulin for 10–15 minutes. Levels were normalized to total protein kinase B (tAkt), i.e., pAkt/tAkt; * p<0.05 Vehicle + Insulin *vs*. Vehicle. (C) Representative immunoblot and quantification of pJNK(Thr183/Tyr185) in protein extracts from different doses of PA-treated podocytes. Levels were normalized to JNK-total (pJNK/JNK-total), (n = 2–3 experiments). (D) Representative immunoblot and quantification of p(Ser307)IRS-1 protein extracts from different doses of PA-treated podocytes. Data is expressed as mean ± SEM and levels were normalized to total IRS-1 (p(Ser307)IRS-1/tIRS-1); *** p<0.001 PA *vs*. Veh.; ** p<0.01 PA *vs*. Veh.; * p<0.05 PA *vs*. Veh.

The mRNA expression of genes involved in glucose metabolism and insulin signaling were also analyzed in podocytes treated with different doses of PA (Fig 3A). Pyruvate carboxylase (PC) enzyme and *Irs-2* mRNA expression showed a significant decrease in podocytes treated with doses of 500 or 750 μM of PA compared to vehicle. *Irs-1* gene expression also decreased significantly at 500 μM of PA. At the same dose of PA, the glucose transporter *GLUT-1* gene expression increased significantly compared to vehicle. However, *GLUT-4* gene expression did not show significant changes at the PA doses used in this study. Finally, *Tlr-4* gene expression also showed no significant change when comparing podocytes treated with PA to podocytes treated with vehicle.

Next, we studied the PI3K/PKB (or Akt) signaling pathway. Podocytes were treated with vehicle, 500 or 750 μM of PA for 24 h, and 100 nM of insulin for 5–10 min. An increase in Akt phosphorylation signal (pAkt) was observed in vehicle-treated podocytes in the presence of insulin (Fig 3B). In contrast, podocytes treated with a dose of 500 or 750 μM of PA did not show this increase in the presence of insulin. As serine phosphorylation has been demonstrated to be a mechanism by which insulin signaling is attenuated, we also explored IRS-1 serine 307 phosphorylation. Fig 3D shows an increase of IRS-1 serine 307 phosphorylation (p(Ser307)IRS-1) in 750 μM PA-treated podocytes compared to vehicle-treated podocytes.

Furthermore, activation of JNK has been implicated in phosphorylating the IRS1 Ser307 residue in insulin resistant mice [37]. Fig 3C shows an increased JNK phosphorylation in podocytes treated with 500 or 750 μM of PA compared to vehicle-treated podocytes.

### Oxidative stress and endoplasmic reticulum stress in insulin-resistant podocytes in the presence of PA

**Fig 4:**
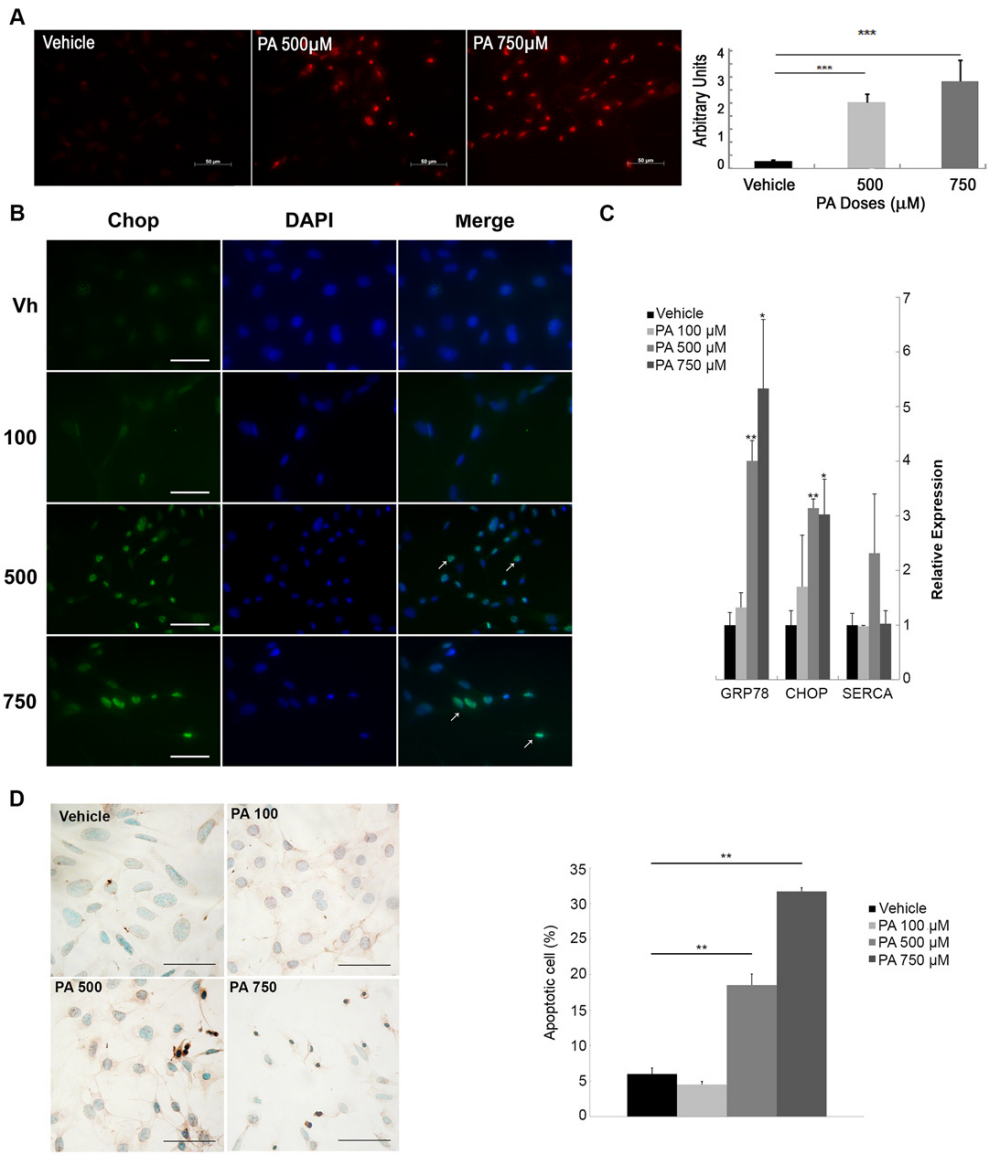
PA treatment induces oxidative and ER stress and is associated with apoptosis in podocytes. (A) Representative photographs of the nuclei of podocytes treated with vehicle, 100, 500 or 750 μM of PA, then incubated with Di-hydroethidium (DHE) probe and red fluorescence quantification is expressed in arbitrary units (A.U.) (n = 3 experiments). Original magnification: 200×. (B) Representative micrographs of the immunofluorescence for c/EBP homologous protein (CHOP) in podocytes treated 24 h with vehicle, 100, 500 or 750 μM of PA (white arrows at stained nuclei). (C) mRNA levels of endoplasmic reticulum stress-related genes such as: *glucose regulated protein 78* (GRP78) and CHOP, *sarcoplasmic reticulum Ca* ^*2+*^ *-ATPase* (SERCA) in podocytes treated 24 h with vehicle, 100, 500 or 750 μM of PA. (D) Representative micrographs of TUNEL assay and quantification of apoptosis in podocytes treated with PA. Data is expressed as mean ± SEM and normalized with GeNorm; *** p<0.001 PA *vs*. Veh.; ** p<0.01 PA *vs*. Veh.; * p<0.05 PA *vs*. Veh.

Release of reactive oxygen species (ROS) is frequently used as an indicator of oxidative stress in cells under insulin resistance states. Fig 4A shows representative photographs of the podocyte nucleus with and without 500 or 750 μM of PA treatment for 24 h. Red fluorescence quantification was significantly increased in podocytes treated with 500 or 750 μM doses of PA compared to podocytes treated with vehicle (Fig 4A).

In addition to oxidative stress, the ER stress response could also be linked to insulin resistance in podocytes [38]. In our study, mRNA expression of the *c/EBP homologous protein (CHOP)* and *glucose regulated protein 78 (GRP78)* increased significantly in podocytes treated with 500 or 750 μM PA. The ER stress response was also confirmed by immunofluorescence for CHOP, with increased CHOP nuclear localization in podocytes treated with 500 or 750 μM of PA compared with vehicle (Fig 4B). The mRNA expression levels of a Ca^2+^-dependent transporter involved in the ER process such as *sarcoplasmic reticulum Ca* ^*2+*^ *-ATPase (SERCA)* did not show significant changes at any dose of PA compared to vehicle-treated cells (Fig 4C). Analysis of western blot of spliced XBP1 and Atg7 mRNA levels did not show ER stress associated with autophagy (S1 Fig).

Associated with the oxidative stress and ER stress, apoptosis determined by TUNEL was also found in PA-treated podocytes with DNA becoming more fragmented as the PA dose increased (Fig 4D). Moreover, mRNA levels of genes related to apoptosis such as Bcl-2 were also decreased in PA-treated podocytes (S1 Fig). However, palmitic acid did not cause increased mRNA expression of genes related to epithelial-mesenchymal transition (EMT) (S1 Fig).

### Cytoskeleton changes associated with insulin resistance in palmitic acid-treated podocytes

**Fig 5:**
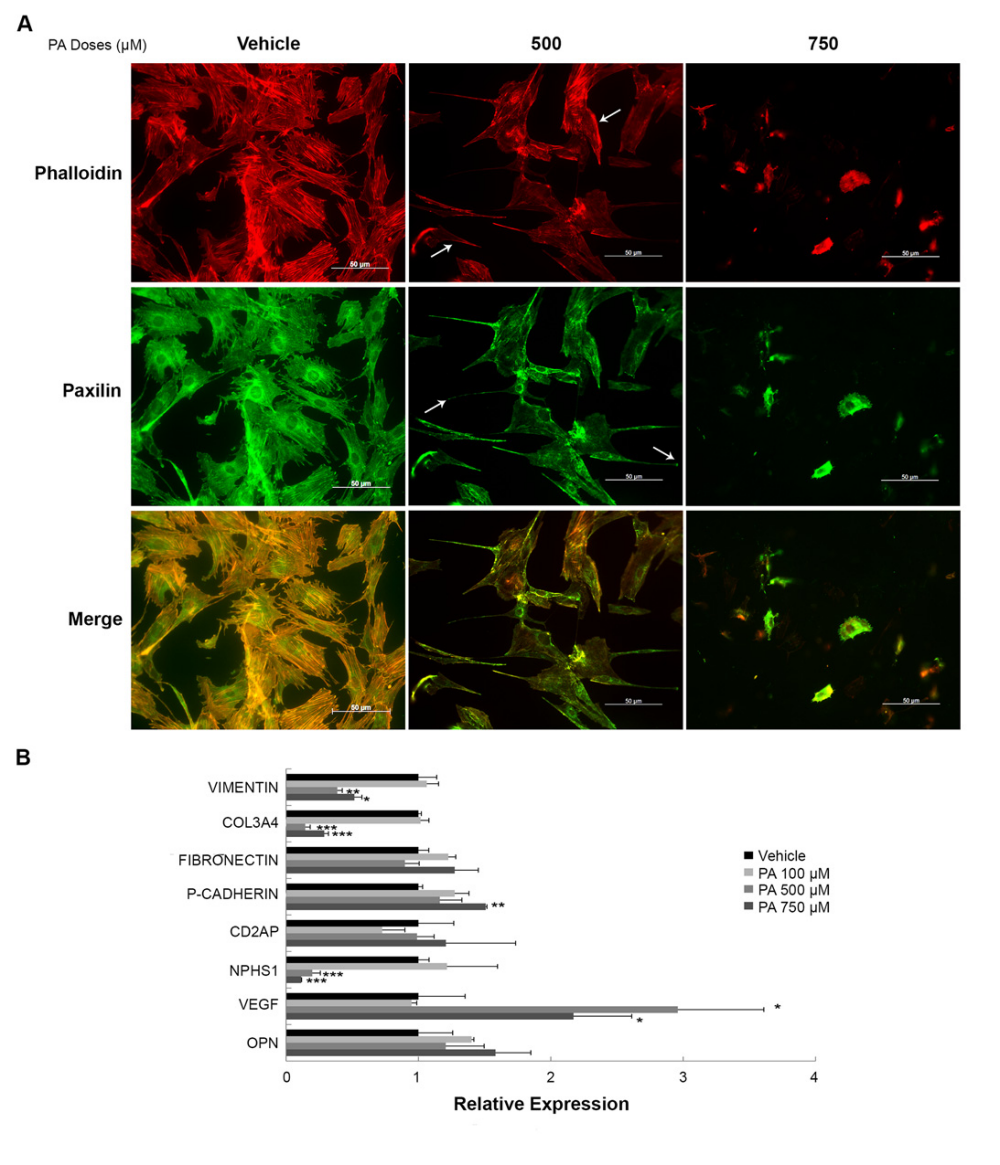
Changes in cytoskeleton and slit-diaphragm related to PA treatment. (A) Representative immunofluorescence micrographs of phalloidin (red) and paxillin (green) staining. White arrows indicate locations of actin filaments (anti-phalloidin) or focal adhesions (anti-paxillin). Original magnification: 200×. (B) mRNA levels of cytoskeleton and slit-diaphragm related genes such as: *Vimentin*, *Collagen3A4* (Col3A4), *Fibronectin* (FN), *P-Cadherin (P–Cad)*, *cluster of differentiation-2 associated protein* (CD2AP), *Nephrin* (Nphs1), *Vascular endothelial growth factor* (VEGF), and *Osteopontin* (OPN) in podocytes treated 24 h with vehicle, 100, 500 or 750 μM of PA. Data is expressed as mean ± SEM and normalized with GeNorm; *** p<0.001 PA *vs*. Veh.; ** p<0.01 PA *vs*. Veh.; * p<0.05 PA *vs*. Veh.

To assess the cytoskeletal structure and its role in the development of insulin resistance in podocytes treated with PA, we performed immunofluorescence (IF) wherein the different components of the cytoskeleton were labelled (Fig 5A). Phalloidin toxin (red colour) was used to label the actin cytoskeleton. As shown in the figure, the actin filaments of podocytes treated with vehicle were arranged radially along the entire cell. However, the actin filaments in the 500 μM PA-treated podocytes were reorganized from the radial area to the peripheral zone. A cytoskeleton breakdown was observed in the 750 μM PA-treated podocytes consistent with the higher level of apoptosis observed. The inadequate polymerization of cytoskeletal actin filaments causes the stress fiber formation and fiber termination in focal adhesion proteins. To visualize this process we performed an anti-paxillin IF (green colour) to label the focal adhesions (Fig 5A). Podocytes treated with 500 or 750 μM of PA showed a larger increase in focal adhesions and stress fiber labelling than in the vehicle-treated podocytes.

We also analyzed changes in gene expression of actin cytoskeleton-related proteins such as the intermediate filament vimentin, collagen3A4 (col3A4) and fibronectin. Analysis of vimentin and col3A4 showed that the mRNA levels of both proteins decreased in podocytes treated with 500 or 750 μM of PA. However, levels of fibronectin did not change at any of the doses used (Fig 5B).

Furthermore, we studied the gene expression of some of the slit-diaphragm proteins involved in adjacent podocyte intercellular junctions (Fig 5B). RT-PCR analysis showed the mRNA expression of *P-Cadherin* to be significantly increased at 750 μM of PA. Cluster of differentiation-2 associated protein (*Cd2ap)* levels showed no statistical differences at any dose of PA used. However, *Nephrin* (Nphs1) gene expression significantly decreased in 500 or 750 μM PA-treated podocytes compared to vehicle (Fig 5B). *Podocin* mRNA levels were undetectable. The angiogenic factor VEGF was also studied. PA-treated podocytes underwent an increase in *Vegf* mRNA levels at the higher doses used, though only reaching statistical significance at 500 μM of PA.

**Fig 6:**
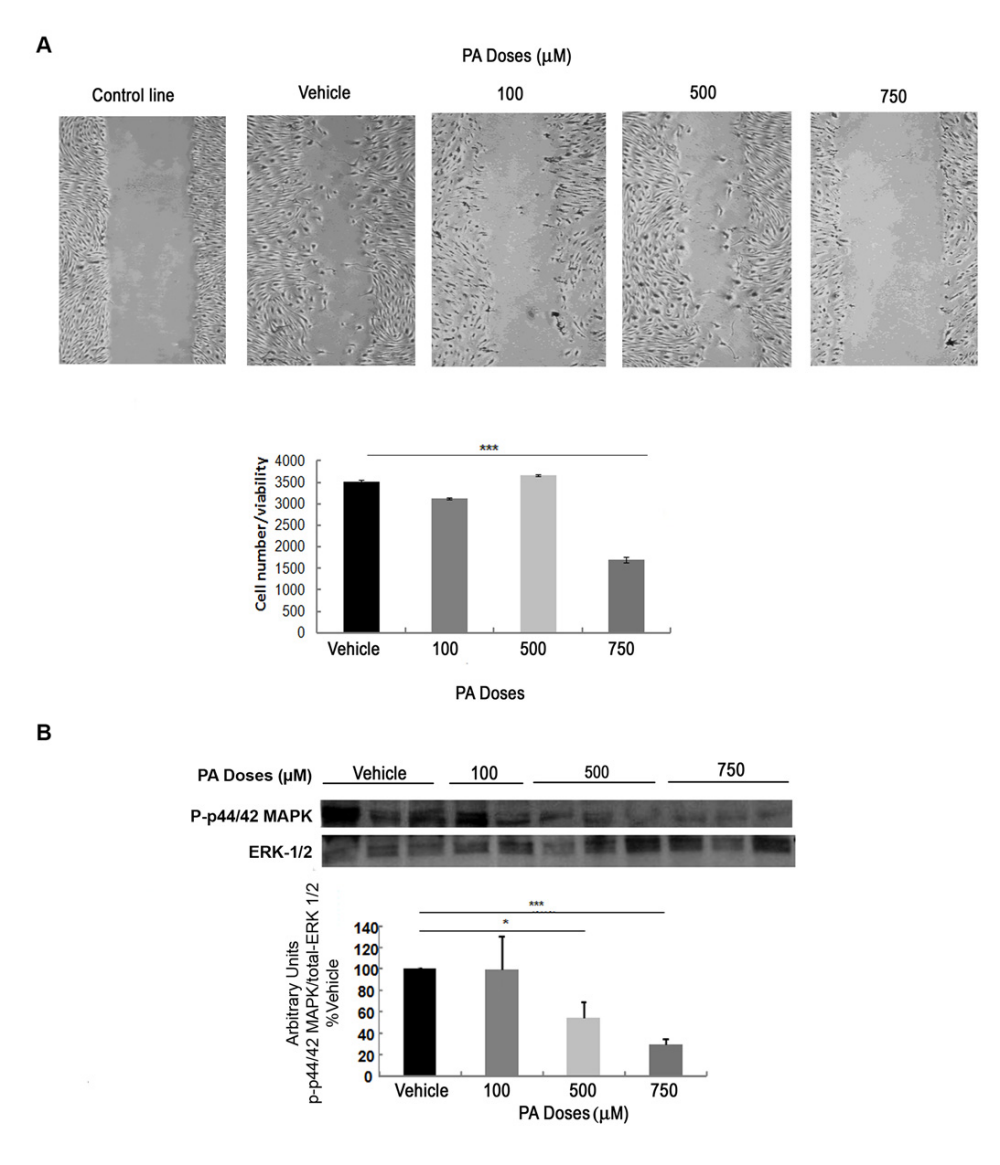
PA produces motility alterations and decreased phosphorylation of p44/42 MAPK in podocytes. (A) Representative micrographs of scratch assays performed on podocytes treated with vehicle, 100, 500 or 750 μM of PA and quantification of the number of migrating cells after 24 h of treatment. Quantification was done based on the negative control. Results were normalized to cell viability data for each well as measured by a Crystal Violet method. Original magnification: 200×. (B) Representative immunoblot and quantification of phospho-p44/42 MAPK in protein extracts from different doses of PA-treated podocytes. Data is expressed as mean ± SEM and levels were normalized to total-ERK1/2 (phospho-p44/42-to-total-ERK1/2 ratio); *** p<0.001 PA *vs*. Veh.; ** p<0.01 PA *vs*. Veh.; * p<0.05 PA *vs*. Veh.

Fig 6A showed changes in cell migration when podocytes were treated with different doses of PA. We found that podocyte motility was not significantly affected at the 100 or 500 μM dose of PA but was significantly changed at the 750 μM dose of PA. At 750 μM of PA we observed a significant decrease in the number of cells that migrated into the scratch compared to the cells treated with vehicle.

We also studied the possible phosphorylation change in p44/42 MAPK protein, as it has been associated with alterations in actin cytoskeletal rearrangements and the PI3K/Akt pathway by other authors [39,40]. Fig 6B shows a significant decrease in p44/42 MAPK phosphorylation in podocytes treated with 500 or 750 μM of PA compared to vehicle. These data suggest that decreased phosphorylation of p44/42 MAPK by accumulation of higher doses of PA might affects cytoskeletal rearrangements and insulin resistance in podocytes.

**Fig 7:**
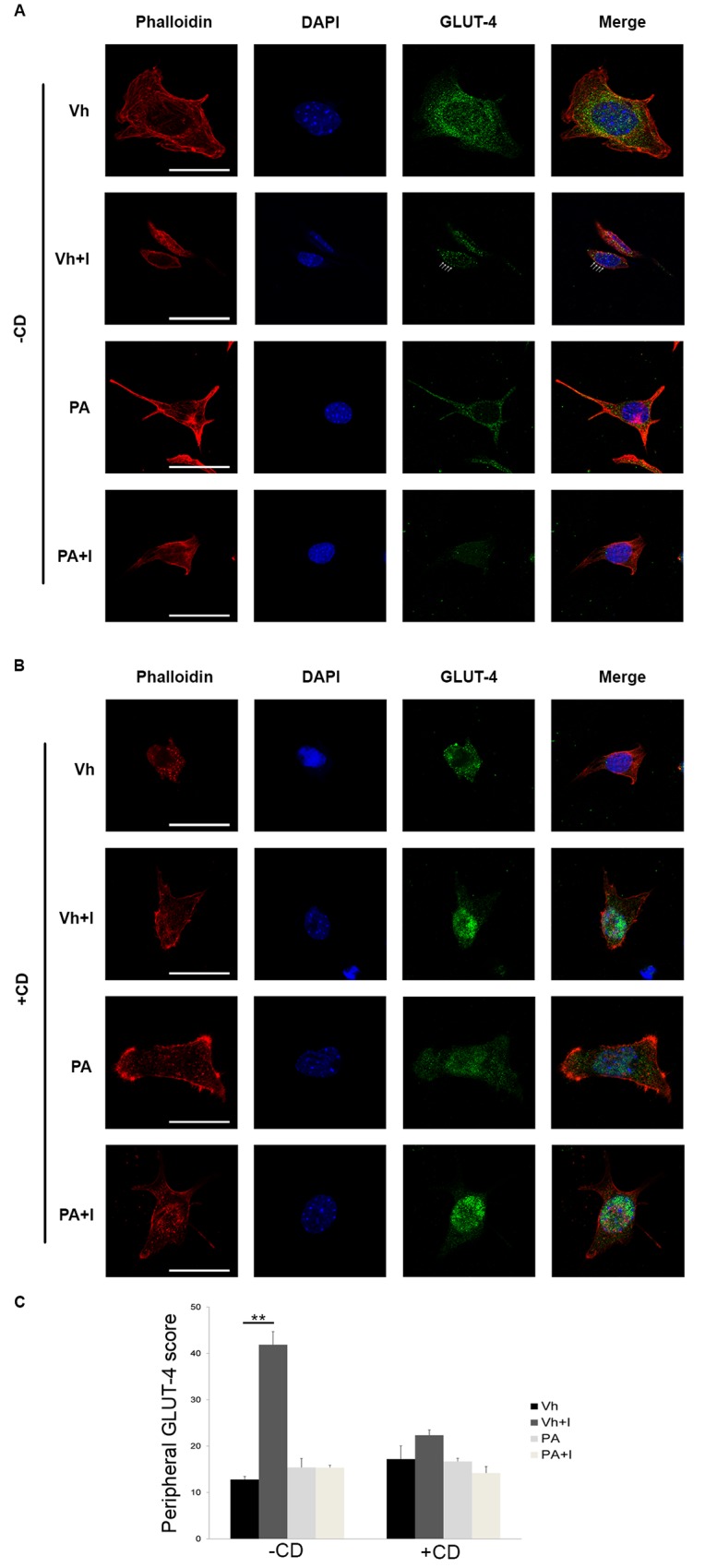
Glucose transporter GLUT4 translocation is dependent on the cytoskeleton in PA-treated podocytes. GLUT4 translocation in response to insulin (100 nM, 10 min) in podocytes treated with vehicle or 500 μM of PA; and (A) untreated or (B) pre-treated with 5 μM of Cytochalasin D for 2 h. Magnification: 1260×. White arrows show peripheral localization of GLUT-4. (C) Quantification of peripheral localization of GLUT-4. *** p<0.001 PA *vs*. Veh.; ** p<0.01 PA *vs*. Veh.; * p<0.05 PA *vs*. Veh.

We specifically analyzed the insulin response of podocytes in the absence/ presence of cytochalasin D (CD), which inhibits actin polymerization and affects cellular motility [41]. Fig 7A and 7B show that CD prevented the insertion of GLUT4 into the membrane under basal conditions. Similarly, GLUT4 remained in the cytoplasm, without insertion into the membrane, when the podocytes were treated with PA.

Of note, CD did not abrogate the phosphorylation of Akt under basal conditions, although this phosphorylation was abrogated in podocytes treated with PA with and without CD (S2 Fig).

## Discussion

In recent years, development of renal disease associated with obesity has emerged as a new concept in the epidemic of Metabolic Syndrome (MetS). In this context, the podocyte has proved to be a key piece in the development of the renal pathology puzzle. Recent work studying insulin resistance in the podocyte promoted by fatty acids or by the deficit of insulin receptor has revealed the crucial role of podocytes in renal damage. However, it is necessary to broaden the knowledge of podocyte dysfunction during the development of MetS. In this study we have investigated the mechanisms that cause podocyte damage by lipid excess. We have found that podocytes treated with PA showed insulin resistance associated with lipid accumulation and an inflammatory state. Moreover, these insulin resistant podocytes exhibited an increase in ROS production and increased ER stress associated with the fatty acid (FA) excess. Increased apoptosis levels were found when increasing doses of PA were used. We also showed a possible role of the cytoskeleton in lipid-induced podocyte dysfunction and insulin resistance, with a cytoskeletal rearrangement and an increase in focal adhesions and stress fibers at high doses of PA.

In this study, podocytes treated with PA showed an accumulation of neutral lipids and a decrease in the gene expression of enzymes involved in *de novo* lipid synthesis. There was also a decrease in FA oxidation. Likewise, we found that the initial step of ceramide synthesis was also decreased. It has been reported that the enzyme involved in the conversion of sphingomyelinase to ceramide, SMPDL3b, was decreased in renal biopsy samples of patients with focal segmental glomerulosclerosis (FSGS). In fact, it was associated with an increased susceptibility of podocytes to injury under this situation [42]. Therefore, the lipid accumulation that we found in podocytes might be induced by a slowdown in lipid metabolism due to FA overload. The gene expression of the isoform *Pparγ1* showed a tendency to be decreased, though not statistically significant, similar to the decrease of *Pparγ1* expression we showed in the kidney of the lipotoxic POKO mouse model [27]. Moreover, the accumulation of lipids seems to produce metabolic activation of several pathways involved in inflammatory processes [43]. The TNK-α signaling pathway was described as one of the routes that contribute to insulin resistance (IR) in different tissues [44]. IL-6 acts as a pro-inflammatory and insulin desensitizer cytokine in adipose tissue and liver. The chemokine MCP-1 increases in high glucose-treated podocytes [45]. In this study, we detected an increase in these inflammatory markers in podocytes treated with PA. This altered expression pattern of proinflammatory genes may also influence the IR observed in podocytes treated with PA, similar to what occurs in skeletal muscle cells [46]. Furthermore, it is known that an increase in FFAs activates proteins such as JNK kinases involved in inflammation through activation of NF-κB [47]. This activation also happens in patients with proteinuria and nephrotic syndrome [48–50]. In our study, a greater localization of NF-κB in the nuclei of podocytes treated with PA and increased JNK phosphorylation confirm the inflammatory state.

Associated with the lipid accumulation and inflammatory state, insulin resistance also developed in the podocytes treated with PA. We have observed a loss of phosphorylation of Akt in response to insulin, similar to other studies [30], and an increase in JNK phosphorylation. This effect on JNK has also been described in hepatocytes and adipocytes exposed to IR conditions [51,52]. In addition, we found an increase of (Ser307) phosphorylation of IRS-1 protein in podocytes treated with the highest dose of PA, similar to muscle cells, adipose tissue and liver [53–55]. Also, our work showed a decrease in mRNA expression of the IRSs at the two higher doses of PA. This reduction could be associated with insulin-dependent decreased activity of PI3K/Akt, as shown in the *Irs-2* deficient hepatocytes [56,57]. It was published recently that *Vegf* gene expression is impaired in the insulin-resistant PodIRKO mouse and in genetically modified mouse podocyte cultures [58]. *Vegf* expression increased in our podocytes treated with PA, similar to what other authors have observed in hepatic cells (Huh7) treated with a mix of FFAs (palmitic and oleic acids) [59]. According to our results, the *Vegf* increase and the interruption of the insulin pathway at two different levels (PI3K and IRS-1) could contribute to the IR produced by FFA in podocytes.

We detected an increase in ROS production associated with FA excess in the podocytes. ROS increases lead to an increase in the phosphorylation of JNK in podocytes [60]. Furthermore, studies performed with *db/db* mouse podocytes and HepG2 hepatocytes reveal that PA increases ROS levels and JNK activation [52,61], confirming our results. Recently, it has been shown that signaling via the XBP1 branch of the unfolded protein response (UPR) is required for an adaptive ER response in diabetic nephropathy. An impaired insulin signaling secondary to insulin resistance promotes a maladaptive UPR with an impaired spliced XBP1 nuclear translocation and is characterized by ATF6 and CHOP signaling in diabetic nephropathy [38]. It is noteworthy that an excessive increase in saturated FAs may alter the phospholipid composition of plasma membranes, and a decrease in the non-saturated FAs ratio causes ER stress [62,63] and produces membrane fluidity loss [64]. Interestingly, although we did not find spliced XBP1 nuclear translocation, we found increased ER stress chaperone protein, Grp78, and one of the main transcription factors that mediate ER stress-induced apoptosis, CHOP. Besides this, TUNEL analysis confirmed ER stress-associated apoptosis in PA-treated podocytes. We suggest that disparate regulation of the UPR branch through induction of CHOP at the mRNA and protein level and final apoptosis could be a link between glomerular cell dysfunction and insulin resistance when there is lipid excess in podocytes.

Herein we also showed the possible role of the cytoskeleton in lipid-induced podocyte dysfunction. We found several cytoskeletal changes in PA-treated podocytes such as an increased number of focal adhesions and stress fibers and a disorganization of actin filaments. A role for the cytoskeleton in podocyte dysfunction has been shown [30]. We also found a radial arrangement of actin filaments and decreased expression of the *col3A4* and *vimentin* genes. Only the gene expression of the slit-diaphragm protein Nphs1 was greatly diminished in this study. *Cd2a*p gene expression did not change in our study, in contrast to what was shown in other studies of nephrotic syndrome patients [65]. However, P-cadherin gene expression tended to increase, perhaps to compensate for the nephrin deficiency. The migration of podocytes is functionally linked to proteinuria and mechanistically linked to the actin cytoskeleton [66]. We found that cell motility only decreases at the highest dose of PA, probably due to apoptosis and cytoskeletal disorganization associated with higher degrees of inflammation, ER and oxidative stress, and insulin resistance in podocytes. An increase in motility has been proposed as a mechanism for podocytopenia in early DN [67]. But if the cytoskeletal damage is prolonged over time, contractility and motility could decrease, as is thought to happen in cases of FSGS [11].

Interestingly, we found a decreased phosphorylation of p44/42 MAPK protein in podocytes treated with 500 or 750 μM doses of PA, suggesting a disruption in the polymerization of actin filaments associated with the abrogation of both MAPK and PI3K signaling pathways in PA-treated podocytes, as previously shown in mice with specific deletion of the insulin receptor from their podocytes [40].

In addition, in human intestinal epithelial cells (HT-29 or Caco-2), disruption of the actin cytoskeleton with cytochalasin D induced the activation of inflammation pathways, demonstrating the concomitant activation of certain pathways resulting from actin disassembly [68]. We found that insulin resistance in the podocyte was associated with secretion of MCP-1 and the gene expression of *tnf-α*, which is also implicated in the peripheral reorganization of actin fibers and increased stress in podocytes [34]. It is known that PA or MCP-1 treatment produces changes at the podocyte cytoskeletal level, leading to alterations in cell motility [11,34]. Thus, under induced FA excess, the increased inflammatory state would be related to not only loss of function of the insulin signaling pathway but also cytoskeleton rearrangement in podocytes. Along this line, it has been described that nephrin participates in the translocation of GLUT4 vesicles to the plasma membrane in human podocytes [30]. We have shown that treatment of podocytes with PA altered the transport of GLUT4 and that the alteration was related to cytoskeleton breakdown. We speculate that altered *Nphs1* expression and cytoskeleton disruption produce an alteration in glucose transport that would reach the threshold of IR.

We propose that there is an important link between the cytoskeleton-to-slit diaphragm complex and insulin signaling in the podocyte. Our experiments show that PA promotes IR, associated with inflammation and parallel to the loss of cytoskeleton organization. In our experiments with cytochalasin D, the podocytes exhibited a derangement of cytoskeletal architecture, which prevented migration of GLUT4 but did not abrogate the Akt phosphorylation. However, the podocytes treated with only PA or cytochalasin D plus PA did not show phosphorylation of Akt in response to insulin. All together, we have shown that PA has a dual effect contributing to the development of IR: abrogating the Akt phosphorylation in response to insulin and promoting disorganization of the cytoskeleton that probably prevents GLUT4 migration to the plasma membrane. It is known that an increase of saturated FAs and a decrease in non-saturated FAs plus cholesterol produce membrane fluidity loss [64]. We speculate that this membrane fluidity change may affect cell signaling and also contribute to the IR situation observed in PA-treated podocytes in our study. Furthermore, changes in ceramide levels affect the biophysical properties of membrane lipid rafts. This is important in receptor trafficking processes, for signaling molecules such as Akt [69] and for slit-diaphragm maintenance [7]. Taken together, we suggest that alterations in slit-diaphragm and cytoskeleton aggravate the insulin resistance induced by lipids and targeting these structures could ameliorate the IR state in podocytes. We propose that strategies aimed at stabilizing the podocyte cytoskeleton and podocyte-podocyte contacts would be crucial for renoprotection under conditions of lipid excess.

In conclusion, we have demonstrated that PA produces lipid accumulation, inflammation, oxidative stress, ER stress and cytoskeleton changes associated with IR driving podocytes to apoptosis. In this context, we have shown at the glomerular level that podocytes are some of the key cells that are damaged due to lipid overload under lipotoxic conditions. Considering that lipid excess plays an important role in triggering IR, we further show that IR manifests as disruptions to the cytoskeleton and slit diaphragm proteins in the podocyte. The interplay of intracellular lipid metabolism, chronic inflammation, insulin resistance and cytoskeletal rearrangements might be important in the pathogenesis of microvascular complications of metabolic diseases.

## Supporting Information

S1 Fig PA treatment produces apoptosis but not EMT or autophagy in cultured podocytes. (A) mRNA levels of apoptosis and EMT-related genes such as: *B-cell CLL/ lymphoma 2* (BCL-2), *Fibroblast growth factor-1* (FGF1), *Cyclin D3*, *transforming growth factor-β1* (TGF-β1), *SNAIL*, *or smooth muscle α-actin* (α-SMA) in podocytes treated 24 h with vehicle, 100, 500 or 750 μM of PA. Data is expressed as mean ± SEM and normalized with GeNorm; (B) mRNA levels of autophagy-related genes such as: *autophagy related gene 7* (ATG7) or *Toll-like Receptor 9 (TLR9)* in podocytes treated 24 h with vehicle, 100, 500 or 750 μM of PA. Data is expressed as mean ± SEM and normalized with GeNorm; (C) Representative immunoblot and quantification showing nuclear and cytoplasmic levels of sXBP1 from different doses of PA-treated podocytes. Nuclear levels were normalized to Lamin B Receptor and cytoplasmic levels to Actin. *** p<0.001 PA *vs*. Veh.; ** p<0.01 PA *vs*. Veh.; * p<0.05 PA *vs*. Veh.
(TIF)

S2 Fig p-Akt/ total-Akt in response to insulin and Cytochalasin D. Representative immunoblot of p-Akt/ total-Akt in response to insulin (100 nM, 5–10 mins) from differentiated podocytes treated with vehicle or 500 μM of PA and untreated (-CD) or treated (+CD) with 5 μM of Cytochalasin D for 2 h. Levels were normalized to total protein kinase B (tAkt) (i.e., pAkt/ tAkt). Data is expressed as mean ± SEM. *** p<0.001 Veh. *vs*. Veh+insulin; ** p<0.01 Veh *vs*. Veh+insulin; * p<0.05 Veh. *vs*. Veh+insulin, with or without CD.
(TIF)

S1 Table Specific primer sequences used in RT-PCR assays. (TIF)

## References

[1] Wild. Estimates for the year 2000 and projections for 2030. World Health. 2004;27: 1047–1053. 10.2337/diacare.27.5.1047 15111519

[2] Haffner SM. Risk Constellations in Patients with the Metabolic Syndrome: Epidemiology, Diagnosis, and Treatment Patterns. Am J Med. Elsevier; 2015;119: S3–S9. 10.1016/j.amjmed.2006.01.008 16563945

[3] Moorhead JF, El-Nahas M, Chan MK, Varghese Z. Lipid nephrotoxicity in chronic progressive glomerular and tubulo-interstitial disease. Lancet. Elsevier; 2015;320: 1309–1311. 10.1016/S0140-6736(82)91513-6 6128601

[4] Ruan XZ, Moorhead JF, Fernando R, Wheeler DC, Powis SH, Varghese Z. Regulation of lipoprotein trafficking in the kidney: role of inflammatory mediators and transcription factors. Biochem Soc Trans. 2004;32: 88–91. 10.1042/BST0320088 14748720

[5] Ke Wf. Lipids and the kidney. Kidney Int. 1994;46: 910–20. 7996813 10.1038/ki.1994.349

[6] Ziyadeh FN. Mediators of diabetic renal disease: the case for tgf-Beta as the major mediator. J Am Soc Nephrol. 2004;15 Suppl 1: S55–S57. 10.1097/01.ASN.0000093460.24823.5B 14684674

[7] Merscher S, Fornoni A. Podocyte pathology and nephropathy—sphingolipids in glomerular diseases. Frontiers in Endocrinology. 2014 10.3389/fendo.2014.00127 25126087 PMC4115628

[8] Braissant O, Wahli W. Differential Expression of Peroxisome Proliferator-Activated Receptor-α, -β, and -γ during Rat Embryonic Development. Endocrinology. The Endocrine Society; 1998;139: 2748–2754. 10.1210/endo.139.6.6049 9607781

[9] Lee HS, Kim BC, Hong HK, Kim YS. LDL stimulates collagen mRNA synthesis in mesangial cells through induction of PKC and TGF-beta expression. Am J Physiol. 1999;277: F369–F376. 10484520 10.1152/ajprenal.1999.277.3.F369

[10] Joles J a, Kunter U, Janssen U, Kriz W, Rabelink TJ, Koomans H a, et al Early mechanisms of renal injury in hypercholesterolemic or hypertriglyceridemic rats. J Am Soc Nephrol. 2000;11: 669–683. 10752526 10.1681/ASN.V114669

[11] Welsh GI, Saleem M a. The podocyte cytoskeleton—key to a functioning glomerulus in health and disease. Nat Rev Nephrol. Nature Publishing Group; 2011;8: 14–21. 10.1038/nrneph.2011.151 22025085

[12] Brown EJ, Schlöndorff JS, Becker DJ, Uscinski AL, Higgs HN, Henderson JM, et al NIH Public Access. 2010;42: 72–76. 10.1038/ng.505.Mutations PMC2980844 20023659

[13] Machuca E, Benoit G, Antignac C. Genetics of nephrotic syndrome: Connecting molecular genetics to podocyte physiology. Hum Mol Genet. 2009;18: 185–194. 10.1093/hmg/ddp328 19808795

[14] Leeuwis JW, Nguyen TQ, Dendooven A, Kok RJ, Goldschmeding R. Targeting podocyte-associated diseases. Adv Drug Deliv Rev. Elsevier B.V.; 2010;62: 1325–1336. 10.1016/j.addr.2010.08.012 20828590

[15] Mundel P, Reiser J, Zúñiga Mejía Borja a, Pavenstädt H, Davidson GR, Kriz W, et al Rearrangements of the cytoskeleton and cell contacts induce process formation during differentiation of conditionally immortalized mouse podocyte cell lines. Exp Cell Res. 1997;236: 248–258. 10.1006/excr.1997.3739 9344605

[16] Lavin PJ, Gbadegesin R, Damodaran T V, Winn MP. Therapeutic targets in focal and segmental glomerulosclerosis. Curr Opin Nephrol Hypertens. 2008;17: 386–392. 10.1097/MNH.0b013e32830464f4 18660675 PMC2674376

[17] Chuang PY, He JC. Signaling in Regulation of Podocyte Phenotypes. Nephron Physiol. 2009;111: p9–15. 10.1159/000191075 19142027 PMC2881215

[18] Kwoh C, Shannon MB, Miner JH, Shaw A. PATHOGENESIS OF NONIMMUNE GLOMERULOPATHIES. Annu Rev Pathol Mech Dis. Annual Reviews; 2006;1: 349–374. 10.1146/annurev.pathol.1.110304.100119 18039119

[19] Ha T-S, Hong E-J, Han G-D. Diabetic conditions downregulate the expression of CD2AP in podocytes via PI3-K/Akt signalling. Diabetes Metab Res Rev. 2015;31: 50–60. 10.1002/dmrr.2562 24846128

[20] Mundel P, Shankland SJ. Podocyte biology and response to injury. J Am Soc Nephrol. 2002;13: 3005–3015. 10.1097/01.ASN.0000039661.06947.FD 12444221

[21] Pavenstädt H, Kriz W, Kretzler M. Cell biology of the glomerular podocyte. Physiol Rev. 2003;83: 253–307. 10.1152/physrev.00020.2002 12506131

[22] Li X, Zhang X, Li X, Ding F, Ding J. The Role of Survivin in Podocyte Injury Induced by Puromycin Aminonucleoside. Int J Mol Sci. 2014;15: 6657–6673. 10.3390/ijms15046657 24747598 PMC4013653

[23] Bijian K, Takano T, Papillon J, Le Berre L, Michaud J-L, Kennedy CRJ, et al Actin cytoskeleton regulates extracellular matrix-dependent survival signals in glomerular epithelial cells. Am J Physiol Renal Physiol. 2005 10.1152/ajprenal.00106.2005 16014575

[24] Ichimura K, Kurihara H, Sakai T. Actin filament organization of foot processes in vertebrate glomerular podocytes. Cell Tissue Res. 2007;329: 541–557. 10.1007/s00441-007-0440-4 17605050

[25] Ichimura K, Kurihara H, Sakai T. Actin filament organization of foot processes in rat podocytes. J Histochem Cytochem. United States; 2003;51: 1589–1600. 14623927 10.1177/002215540305101203

[26] Coward RJM, Welsh GI, Yang J, Tasman C, Lennon R, Koziell A, et al The human glomerular podocyte is a novel target for insulin action. Diabetes. 2005 10.2337/diabetes.54.11.3095 16249431

[27] Martinez-Garcia C, Izquierdo A, Velagapudi V, Vivas Y, Velasco I, Campbell M, et al Accelerated renal disease is associated with the development of metabolic syndrome in a glucolipotoxic mouse model. Disease Models & Mechanisms. 2012. 10.1242/dmm.009266 PMC3424461 22773754

[28] Jat PS, Noble MD, Ataliotis P, Tanaka Y, Yannoutsos N, Larsen L, et al Direct derivation of conditionally immortal cell lines from an H-2Kb-tsA58 transgenic mouse. Proc Natl Acad Sci U S A. 1991;88: 5096–5100. 10.1073/pnas.88.12.5096 1711218 PMC51818

[29] Schmitz-Peiffer C, Craig DL, Biden TJ. Ceramide generation is sufficient to account for the inhibition of the insulin-stimulated PKB pathway in C2C12 skeletal muscle cells pretreated with palmitate. J Biol Chem. 1999;274: 24202–24210. 10.1074/jbc.274.34.24202 10446195

[30] Lennon R, Pons D, Sabin MA, Wei C, Shield JP, Coward RJ, et al Saturated fatty acids induce insulin resistance in human podocytes: Implications for diabetic nephropathy. Nephrol Dial Transplant. 2009 10.1093/ndt/gfp302 PMC7614380 19556298

[31] Daniela Macconi and GR. Permselective dysfunction of podocyte-podocyte contact upon angiotensin II unravels the molecular target for renoprotective intervention. Am J Pathol. 2006;168: 1073–85. 16565484 10.2353/ajpath.2006.050701 PMC1606571

[32] Vivas Y, Martinez-Garcia C, Izquierdo A, Garcia-Garcia F, Callejas S, Velasco I, et al Early Peroxisome proliferator-activated receptor gamma regulated genes involved in expansion of pancreatic beta cell mass. BMC Med Genomics. BioMed Central Ltd; 2011;4: 86 10.1186/1755-8794-4-86 22208362 PMC3315430

[33] MM B. A rapid and sensitive method for the quantitation of microgram quantities of protein utilizing the principle of protein-dye binding. Anal Biochem. 1976;7: 248–54. 10.1016/0003-2697(76)90527-3 942051

[34] Lee EY, Chung CH, Khoury CC, Yeo TK, Pyagay PE, Wang A, et al The monocyte chemoattractant protein-1/CCR2 loop, inducible by TGF-beta, increases podocyte motility and albumin permeability. Am J Physiol Renal Physiol. 2009;297: F85–F94. 10.1152/ajprenal.90642.2008 19420107 PMC2711714

[35] Xaus J, Comalada M, Cardó M, Valledor AF, Celada A. Decorin inhibits macrophage colony-stimulating factor proliferation of macrophages and enhances cell survival through induction of p27Kip1 and p21Waf1. Blood. 2001;98: 2124–2133. 10.1182/blood.V98.7.2124 11567999

[36] Jiménez-Altayó F, Briones a. M, Giraldo J, Planas a. M, Salaices M, Vila E. Increased Superoxide Anion Production by Interleukin-1 beta Impairs Nitric Oxide-Mediated Relaxation in Resistance Arteries. J Pharmacol. 2006;316: 42–52. 10.1124/jpet.105.088435.to 16183707

[37] Ozcan U, Yilmaz E, Ozcan L, Furuhashi M, Vaillancourt E, Smith RO, et al Chemical chaperones reduce ER stress and restore glucose homeostasis in a mouse model of type 2 diabetes. Science. United States; 2006;313: 1137–1140. 10.1126/science.1128294 16931765 PMC4741373

[38] Madhusudhan T, Wang H, Dong W, Ghosh S, Bock F, Thangapandi VR, et al Defective podocyte insulin signalling through p85-XBP1 promotes ATF6-dependent maladaptive ER-stress response in diabetic nephropathy. Nat Commun. Nature Publishing Group; 2015;6: 6496 10.1038/ncomms7496 25754093 PMC4366504

[39] Crean JKG, Finlay D, Murphy M, Moss C, Godson C, Martin F, et al The role of p42/44 MAPK and protein kinase B in connective tissue growth factor induced extracellular matrix protein production, cell migration, and actin cytoskeletal rearrangement in human mesangial cells. J Biol Chem. 2002;277: 44187–44194. 10.1074/jbc.M203715200 12218048

[40] Welsh GI, Hale LJ, Eremina V, Jeansson M, Maezawa Y, Lennon R, et al Insulin signaling to the glomerular podocyte is critical for normal kidney function. Cell Metab. United States; 2010;12: 329–340. 10.1016/j.cmet.2010.08.015 PMC4949331 20889126

[41] Goddettes DW, Frieden C. Actin Polymerization. 1986; 15974–15980. 3023337

[42] Katz H, Fornoni A, Merscher S, Kopp JB. Lipid biology of the podocyte—new perspectives offer new opportunities. Nat Rev Nephrol. 2014;10: 379–388. 10.1038/nrneph.2014.87 24861084 PMC4386893

[43] Kume S, Uzu T, Araki S, Sugimoto T, Isshiki K, Chin-Kanasaki M, et al Role of altered renal lipid metabolism in the development of renal injury induced by a high-fat diet. J Am Soc Nephrol. 2007;18: 2715–2723. 10.1681/ASN.2007010089 17855643

[44] Hivert M-F, Sullivan LM, Fox CS, Nathan DM, D’Agostino RB, Wilson PWF, et al Associations of Adiponectin, Resistin, and Tumor Necrosis Factor-α with Insulin Resistance. J Clin Endocrinol Metab. The Endocrine Society; 2008;93: 3165–3172. 10.1210/jc.2008-0425 18492747 PMC2515087

[45] Nam BY, Paeng J, Kim SH, Lee SH, Kim DH, Kang HY, et al The MCP-1/CCR2 axis in podocytes is involved in apoptosis induced by diabetic conditions. Apoptosis. 2012;17: 1–13. 10.1007/s10495-011-0661-6 22006533

[46] Schenk S, Saberi M, Olefsky JM. Insulin sensitivity: Modulation by nutrients and inflammation. J Clin Invest. 2008;118: 2992–3002. 10.1172/JCI34260 18769626 PMC2522344

[47] Shoelson SE, Lee J, Goldfine AB. Review series Inflammation and insulin resistance. J Clin Invest. 2006;116: 1793–1801. 10.1172/JCI29069.and 16823477 PMC1483173

[48] Thomas ME, Morrison AR, Schreiner GF. Metabolic effects of fatty acid-bearing albumin on a proximal tubule cell line. Am J Physiol—Ren Physiol. 1995;268: F1177–F1184. Available: http://ajprenal.physiology.org/content/268/6/F1177.abstract 10.1152/ajprenal.1995.268.6.F1177 7611458

[49] Thomas ME, Harris KP, Ramaswamy C, Hattersley JM, Wheeler DC, Varghese Z, Williams JD, Walls J MJ. Simvastatin therapy for hypercholesterolemic patients with nephrotic syndrome or significant proteinuria. Kidney Int. 1993;44: 1124–9. 8264145 10.1038/ki.1993.358

[50] Thomas ME, Harris KPG, Walls J, Furness PN, Brunskill NJ. Fatty acids exacerbate tubulointerstitial injury in protein-overload proteinuria. Am J Physiol Renal Physiol. 2002;283: F640–F647. 10.1152/ajprenal.00001.2002 12217854

[51] Gao Z, Zhang X, Zuberi A, Hwang D, Quon MJ, Lefevre M, et al Inhibition of insulin sensitivity by free fatty acids requires activation of multiple serine kinases in 3T3-L1 adipocytes. Mol Endocrinol. 2004;18: 2024–2034. 10.1210/me.2003-0383 15143153

[52] Gao D, Nong S, Huang X, Lu Y, Zhao H, Lin Y, et al The effects of palmitate on hepatic insulin resistance are mediated by NADPH oxidase 3-derived reactive oxygen species through JNK and p38 MAPK pathways. J Biol Chem. 2010;285: 29965–29973. 10.1074/jbc.M110.128694 20647313 PMC2943261

[53] Morino K, Neschen S, Bilz S, Sono S, Tsirigotis D, Reznick RM, et al Muscle-Specific IRS-1 Ser 3 Ala Transgenic Mice Are Skeletal Muscle. 2014;57 10.2337/db06-0454.K.M.

[54] Senn JJ. Toll-like receptor-2 is essential for the development of palmitate-induced insulin resistance in myotubes. J Biol Chem. 2006;281: 26865–26875. 10.1074/jbc.M513304200 16798732

[55] Rotter V, Nagaev I, Smith U. Interleukin-6 (IL-6) Induces Insulin Resistance in 3T3-L1 Adipocytes and Is, Like IL-8 and Tumor Necrosis Factor-α, Overexpressed in Human Fat Cells from Insulin-resistant Subjects. J Biol Chem. 2003;278: 45777–45784. 10.1074/jbc.M301977200 12952969

[56] Valverde AM, González-Rodríguez A. IRS2 and PTP1B: Two opposite modulators of hepatic insulin signalling. Arch Physiol Biochem. 2011;117: 105–115. 10.3109/13813455.2011.557386 21401320

[57] González-rodríguez Á, Más-gutierrez J a, Mirasierra M, Lee YJ, Ko HJ, Kim JK, et al NIH Public Access. 2013;11: 284–296. 10.1111/j.1474-9726.2011.00786.x.ESSENTIAL

[58] Hale LJ, Mathieson W, Welsh GI, Coward RJ, Hurcombe J, Lay A, et al glomerular podocyte Insulin directly stimulates VEGF-A production in the Insulin directly stimulates VEGF-A production in the glomerular podocyte. Am J Physiol Ren Physiol. 2013;305: 182–188. 10.1152/ajprenal.00548.2012 PMC3725664 23698113

[59] Chavez-Tapia NC, Rosso N, Tiribelli C. Effect of intracellular lipid accumulation in a new model of non-alcoholic fatty liver disease. BMC Gastroenterol. BioMed Central Ltd; 2012;12: 20 10.1186/1471-230X-12-20 22380754 PMC3313845

[60] Kamata H, Oka S, Shibukawa Y, Kakuta J HH. Redox regulation of nerve growth factor-induced neuronal differentiation of PC12 cells through modulation of the nerve growth factor receptor, TrkA. Arch Biochem Biophys. 2005;434: 16–25. 15629104 10.1016/j.abb.2004.07.036

[61] Ijaz A, Tejada T, Catanuto P, Xia X, Elliot SJ, Lenz O, et al Inhibition of C-jun N-terminal kinase improves insulin sensitivity but worsens albuminuria in experimental diabetes. Kidney Int. 2009;75: 381–388. 10.1038/ki.2008.559 18971923

[62] Ariyama H, Kono N, Matsuda S, Inoue T, Arai H. Decrease in membrane phospholipid unsaturation induces unfolded protein response. J Biol Chem. 2010;285: 22027–22035. 10.1074/jbc.M110.126870 20489212 PMC2903364

[63] Thörn K, Bergsten P. Fatty acid-induced oxidation and triglyceride formation is higher in insulin-producing MIN6 cells exposed to oleate compared to palmitate. J Cell Biochem. Wiley Subscription Services, Inc., A Wiley Company; 2010;111: 497–507. 10.1002/jcb.22734 20524206

[64] Hagen RM, Rodriguez-Cuenca S, Vidal-Puig A. An allostatic control of membrane lipid composition by SREBP1. FEBS Lett. Federation of European Biochemical Societies; 2010;584: 2689–2698. 10.1016/j.febslet.2010.04.004 20385130

[65] Coward RJM, Foster RR, Patton D, Ni L, Lennon R, Bates DO, et al Nephrotic plasma alters slit diaphragm-dependent signaling and translocates nephrin, Podocin, and CD2 associated protein in cultured human podocytes. J Am Soc Nephrol. 2005;16: 629–637. 10.1681/ASN.2004030172 15659563

[66] Müller-Krebs S, Weber L, Tsobaneli J, Kihm LP, Reiser J, Zeier M, et al Cellular effects of Everolimus and Sirolimus on podocytes. PLoS One. 2013;8: 1–13. 10.1371/journal.pone.0080340 PMC3829970 24260371

[67] Steffes MW. Affecting the decline of renal function in diabetes mellitus. Kidney Int. 2001;60: 378–379. 10.1046/j.1523-1755.2001.00812.x 11422776

[68] Németh ZH, Deitch EA, Davidson MT, Szabó C, Vizi ES, Haskó G. Disruption of the actin cytoskeleton results in nuclear factor-κB activation and inflammatory mediator production in cultured human intestinal epithelial cells. J Cell Physiol. Wiley Subscription Services, Inc., A Wiley Company; 2004;200: 71–81. 10.1002/jcp.10477 15137059

[69] Carobbio S, Rodriguez-Cuenca S, Vidal-Puig A. Origins of metabolic complications in obesity. Curr Opin Clin Nutr Metab Care. 2011;14: 520–526. 10.1097/MCO.0b013e32834ad966 21849895

