# Supplementary figures and images for: Renal Lipotoxicity-Associated Inflammation and Insulin Resistance Affects Actin Cytoskeleton Organization in Podocytes
Source: PLoS One. 2015 Nov 6;10(11):e0142291. doi: 10.1371/journal.pone.0142291 (PMC4636358; doi:10.1371/journal.pone.0142291)

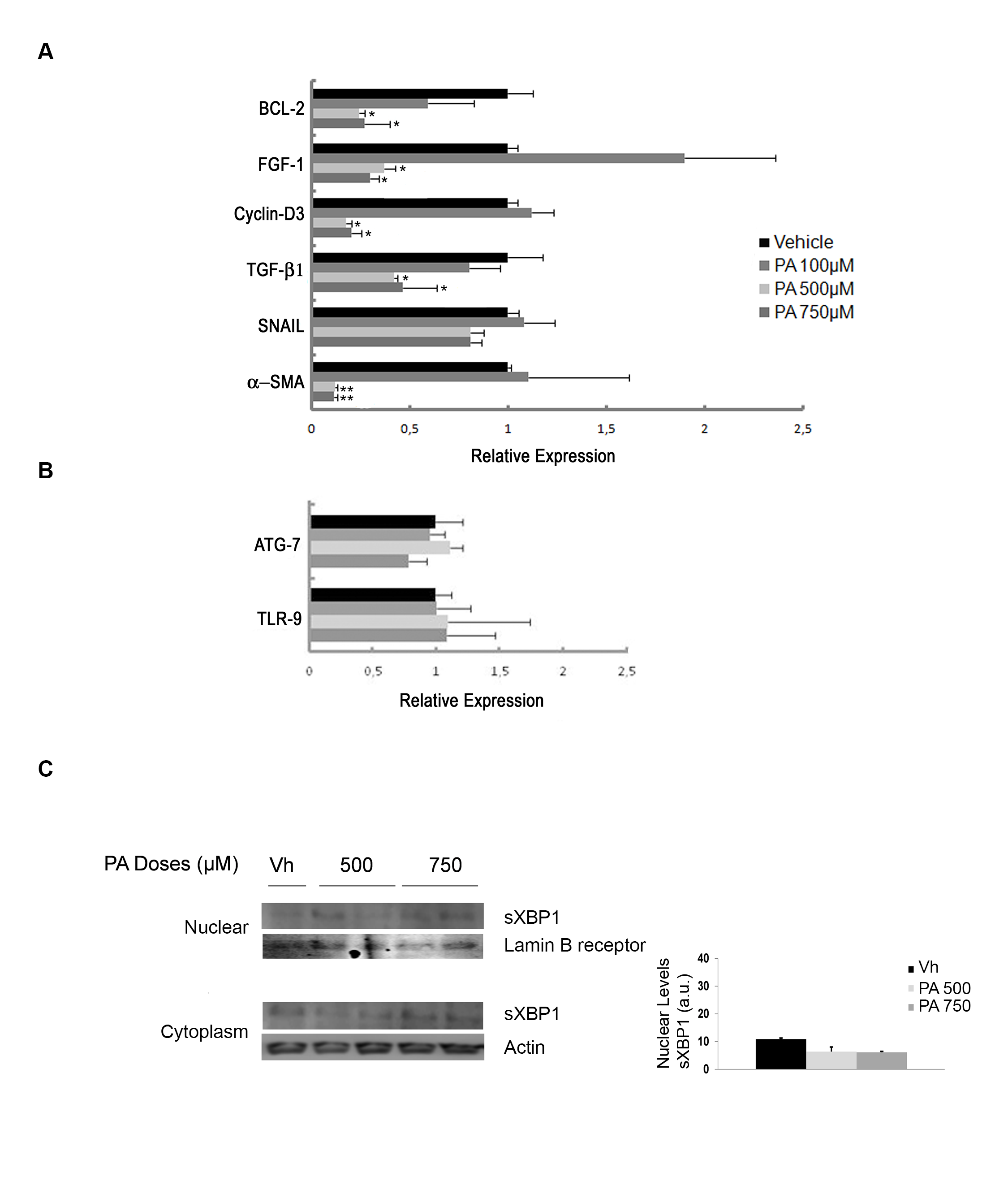

Supplement: S1 Fig — (A) mRNA levels of apoptosis and EMT-related genes such as: B-cell CLL/ lymphoma 2 (BCL-2), Fibroblast growth factor-1 (FGF1), Cyclin D3, transforming growth factor-β1 (TGF-β1), SNAIL, or smooth muscle α-actin (α-SMA) in podocytes treated 24 h with vehicle, 100, 500 or 750 μM of PA. Data is expressed as mean ± SEM and normalized with GeNorm; (B) mRNA levels of autophagy-related genes such as: autophagy related gene 7 (ATG7) or Toll-like Receptor 9 (TLR9) in podocytes treated 24 h with vehicle, 100, 500 or 750 μM of PA. Data is expressed as mean ± SEM and normalized with GeNorm; (C) Representative immunoblot and quantification showing nuclear and cytoplasmic levels of sXBP1 from different doses of PA-treated podocytes. Nuclear levels were normalized to Lamin B Receptor and cytoplasmic levels to Actin. *** p<0.001 PA vs. Veh.; ** p<0.01 PA vs. Veh.; * p<0.05 PA vs. Veh. (TIF) [file pone.0142291.s001.tif]

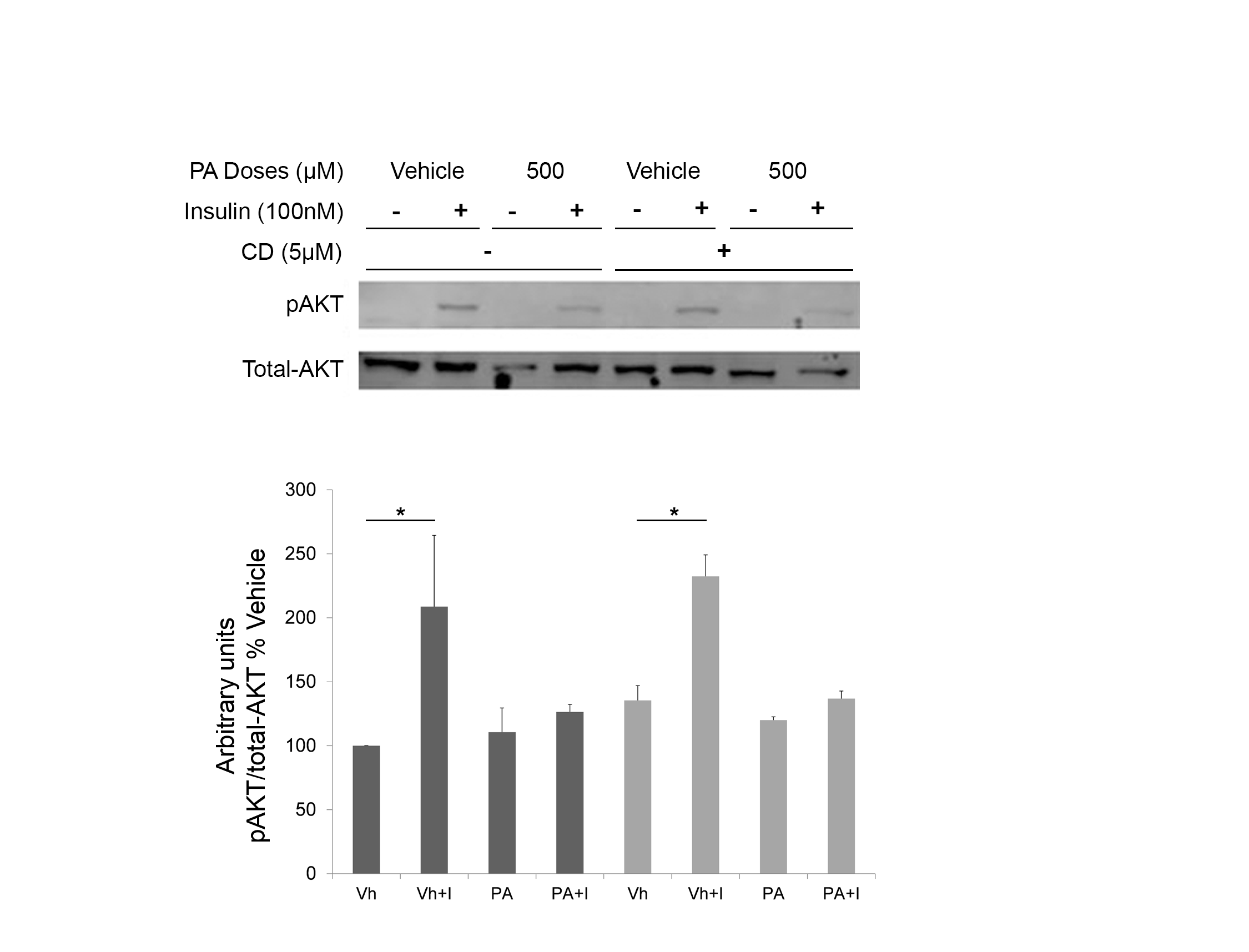

Supplement: S2 Fig — Representative immunoblot of p-Akt/ total-Akt in response to insulin (100 nM, 5–10 mins) from differentiated podocytes treated with vehicle or 500 μM of PA and untreated (-CD) or treated (+CD) with 5 μM of Cytochalasin D for 2 h. Levels were normalized to total protein kinase B (tAkt) (i.e., pAkt/ tAkt). Data is expressed as mean ± SEM. *** p<0.001 Veh. vs. Veh+insulin; ** p<0.01 Veh vs. Veh+insulin; * p<0.05 Veh. vs. Veh+insulin, with or without CD. (TIF) [file pone.0142291.s002.tif]

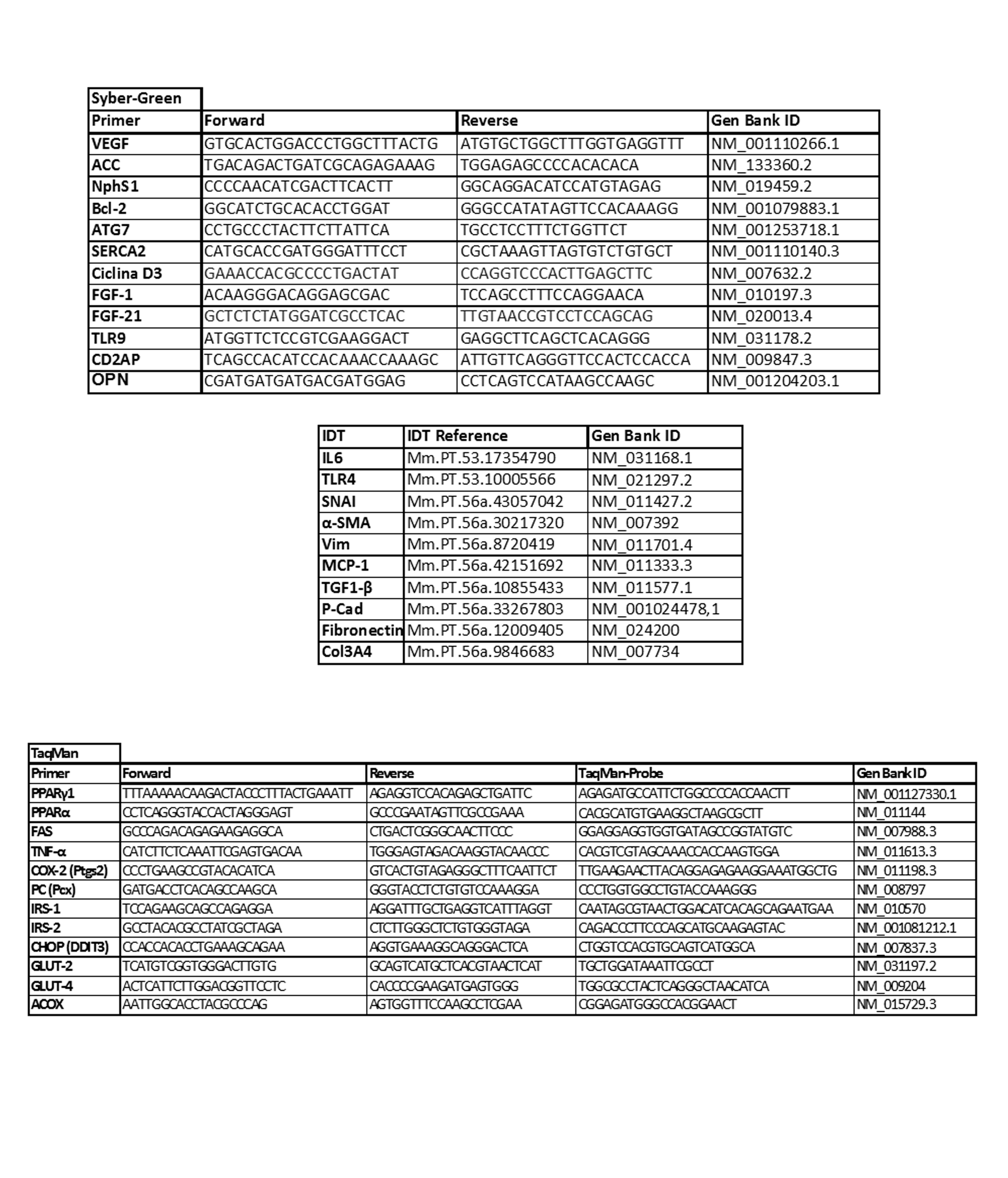

Supplement: S1 Table — (TIF) [file pone.0142291.s003.tif]
